# A Novel Densely Packed 4 × 4 MIMO Antenna Design for UWB Wireless Applications

**DOI:** 10.3390/s23218888

**Published:** 2023-11-01

**Authors:** Owais Khan, Shahid Khan, Safdar Nawaz Khan Marwat, Neelam Gohar, Muhammad Bilal, Mariana Dalarsson

**Affiliations:** 1 Department of Electrical and Computer Engineering, COMSATS University Islamabad, Abbottabad-Campus, Abbottabad 22060, Pakistan; engrowais615@gmail.com (O.K.); shahid@cuiatd.edu.pk (S.K.); 2 Department of Computer Systems Engineering, Faculty of Electrical and Computer Engineering, University of Engineering and Technology Peshawar, Peshawar 25120, Pakistan; safdar@uetpeshawar.edu.pk; 3 Department of Computer Science, Shaheed Benazir Bhutto Women University, Peshawar 25000, Pakistan; neelam.gohar@sbbwu.edu.pk; 4 Department of Information Engineering Technology, University of Technology Nowshera, Nowshera 24100, Pakistan; mbilal@uotnowshera.edu.pk; 5 School of Electrical Engineering and Computer Science, KTH Royal Institute of Technology, SE 100-44 Stockholm, Sweden

**Keywords:** UWB, MIMO, partial ground, ECC, DG, impedance bandwidth

## Abstract

In this article, a compact 4-port UWB (Ultra-Wide Band) MIMO (Multiple Input Multiple Output) antenna is proposed. A low profile FR-4 substrate is used as a dielectric material with the dimensions of 58 × 58 mm^2^ (0.52λ × 0.52λ) at 2.8 GHz and a standard thickness of 1.6 mm. The proposed design characterizes an impedance bandwidth starting from 2.8 to 12.1 GHz (124.1%). Each of the four elements of the proposed MIMO antenna configuration consists of a monopole antenna with PG (partial ground) that has a slot at its center. The corner of each patch (radiator) and ground slot are rounded for impedance matching. Each unit cell is in an orthogonal orientation, forming a quad-port MIMO antenna system. For reference, the partial ground of each unit cell is connected meticulously with the others. The simulated results of the proposed quad-port MIMO antenna design were configured and validated by fabrication and testing. The proposed Quad-port MIMO design has a 6.57 dBi peak gain and 97% radiation efficiency. The proposed design has good isolation below 15 dB in the lower frequency range and below 20 dB in the higher frequency range. The design has a measured ECC (Envelop Correlation Co-efficient) of 0.03 and DG (Diversity Gain) of 10 dB. The value of TARC (Total Active Reflection Coefficient) over the entire operating band is less than 10 dB. Moreover, the design maintained CCL (Channel Capacity Loss) < 0.4 bits/sec/Hz and MEG (Mean Effective Gain) < 3 dB. Based on the obtained results, the proposed design is suitable for the intended high data rate UWB wireless communication portable devices.

## 1. Introduction

The wireless communication industry is currently experiencing a tremendous growth in the area of Radio Frequency (RF) and antenna design. Researchers are trying to find new ways to design antennas with high data rates and low power consumption. Ultra-wideband (UWB) MIMO (Multiple Input Multiple Output) antenna design is an ultimate choice for short-range wireless applications [1,2,3]. For UWB technology, the frequency spectrum, ranging from 3.1 to 10.6 GHz (7.5 GHz bandwidth), has been officially allotted by the Federal Communication Commission (FCC) in USA [4]. Hence, the use of MIMO antenna concepts for UWB technology offers a potential solution for data rate enhancement with low power consumption [5]. Despite having various benefits, MIMO technologies in communication systems are accompanied by some challenges that need proper attention. One of them is to achieve compactness of the overall design while maintaining a good performance. Achieving compactness increases the mutual coupling due to current flow among the ports, which results in poor diversity gain, low radiation efficiency, and low gain. Through proper isolation between the antenna elements, high mutual coupling is avoided. Different methods have been explored and proposed by researchers to enhance isolation among the antenna elements [6]. These techniques include DGS (Defected Ground Structure) [7], stub loading [8,9], NLs (Neutralization lines), isolators with metamaterials [10], and parasitic materials [11,12]. Numerous works have been reported in the literature that simultaneously addresses compactness and mutual coupling [13,14,15,16,17,18,19,20,21,22,23,24,25,26,27,28].

### Literature Review

In [13], a step-shaped slotted MIMO antenna for UWB application is presented. The proposed antenna has overall 42 × 25 mm^2^ dimensions. The operating bandwidth of the design is 3.2–12 GHz with 4 dBi peak gain. The mutual impedance among the antenna elements is 22 dB without using any decoupling mechanism. Although the design is compact with good performance, the drawback is its complex geometry, which makes the fabrication and testing difficult. A compact 4-port slotted MIMO antenna of rectangular shape is reported in [14]. The antenna has dimensions of 39 × 39 mm^2^. The operating bands of the reported design are 3.25–3.75 GHz, 5.08–5.90 GHz, and 7.06–7.95 GHz with a gain varying between 1.4 and 4.6 dBi. Isolation of 22 dB and TARC > −10 dB is achieved using 4-staircase-shaped decoupling techniques. The overall design has a complex structure and decoupling technique. In [15], a 2-port compact CPW-MIMO (Co-planner wave-guide) antenna is presented. The reported antenna has a half-slot structure with an overall dimension of 23 × 18 mm^2^. A Y-shaped slot is introduced at the bottom of the MIMO antenna for isolation enhancement, which results in 15 dB isolation at lower frequencies of the UWB spectrum. A gain varying between 2.55 and 3.58 dBi is achieved over the operating band. The reported design has two elements with complex structures and low port isolation. Another 2-port UWB-MIMO antenna of rectangular shape using CSRR (complementary Split Ring resonator) is reported in [16]. This antenna has an overall size of 30 × 42 × 0.79 mm^3^ with an overall gain between 2 and 5.2 dBi, and 15 dB isolation among ports is achieved. The reported antenna has a band notched with a T-shaped stub in the ground plane. The results of the proposed design show that the designed antenna is not in the UWB frequency range. Moreover, the design exhibits low isolation, which reduces its usefulness for real-time applications. In [17], a quad-port UWB-MIMO antenna that has concentric rings in the resonator with a partial ground is demonstrated. The overall dimension of the antenna is 36 × 36 × 1.6 mm^3^, achieving a 70% efficiency and 15 dB port isolation. The drawback of the reported design is low isolation and a disconnected ground. A planner 4-port UWB-MIMO antenna using a 3 × 3 EBG (Electromagnetic Band Gap) array structure is reported in [18]. The total dimensions of the proposed design are 60 × 60 × 1.6 mm^3^. The reported design resonates at 3–16.2 GHz with a sharp band notch at 4.6 GHz. This design has 17.5 dB isolation with larger dimensions and a disconnected ground. In [19], a 4-element MIMO antenna-based SRR (Split Ring Resonator) is presented. The total size of the system is 136 × 136 mm^2^. The antenna has an overall bandwidth of 2.2–6.28 GHz, covering LTE (2.2–3.8 GHz), Bluetooth (2.4 GHz), WLAN (2.4, 5.1–5.8 GHz), WiMAX (2.3–5.7 GHz), and ISM (2.4/5.2/5.8) bands. However, the reported design has achieved a 4 dBi peak gain and 14 dB port isolation. Larger dimensions and low port isolation reduce the usefulness of the reported design. A 2-port UWB-MIMO antenna with elliptical-shaped radiators is reported in [20]. The reported design has a dimension of 60 × 26 mm^2^. The proposed system operates at 3.1–10.6 GHz with low impedance matching and port isolation. Each of the aforementioned designs makes a compromise between size and achieved results. Some of them are compact in size with low gain and port isolation. Some designs show relatively high performance but are larger in size with complex decoupling techniques, which reduces their usefulness.

From the literature review, we obtained the following useful information:

(i) MIMO antennas are helpful in enhancing the data rate without the provision of additional bandwidth.
(ii) Achieving high performance, high port isolation, and a compact size without additional structure with the design is challenging.

The scientific novelty of the proposed design is driven by the design simplicity and achieved results. The proposed design has a simple structure without the incorporation of complex techniques or additional structures like metamaterials or metallic vias for the decoupling, bandwidth, and gain enhancement. In this work, a compact four-port UWB-MIMO antenna operating at 2.8–12 GHz is reported. The proposed design has a connected ground and a simple decoupling technique. The main contributions of the proposed work are as follows:

- The proposed design has a partial ground with a rectangular slot to offer a wide band with better impedance matching.
- Cross-shaped strips above the substrate are symmetrically added among the MIMO elements to enhance the ports’ isolation and impedance bandwidth.
- The designed antenna is very compact in term of electrical and physical size.
- A high efficiency and Gain is achieved over the entire operating band.
- High isolation has been maintained over the operating band with a novel decoupling technique.

The rest of the article is structured as follows: The suggested antenna design is discussed in Section 2. Section 3 highlights the design configuration and evaluation. Section 4 discusses the simulated and experimental results. Section 5 concludes the paper.

## 2. Proposed Antenna Design

**Figure 1:**
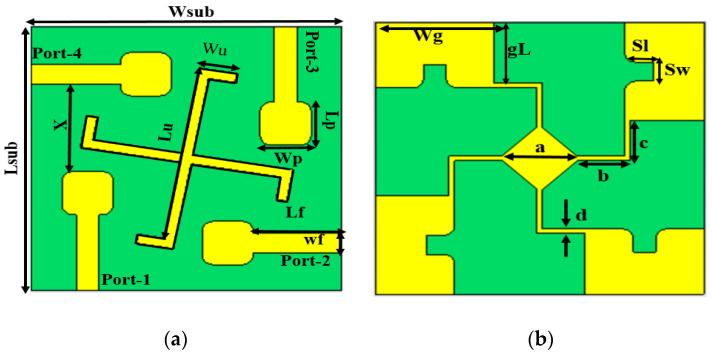
The Proposed 4-port UWB-MIMO antenna: (**a**) top view and (**b**) bottom view.

The proposed 4-port UWB-MIMO antenna incorporates four single-unit cell microstrip patch antennas (MPA). As depicted in Figure 1a, these MIMO elements are placed on the top of the FR4 substrate with a relative permittivity (Ɛr) of 4.3 and a standard thickness of 1.6 mm. These MPAs have similar dimensions and are positioned in an orthogonal orientation on the substrate. W_S_ and L_S_ are the width and length of the substrate, respectively. L_P_ and W_P_ are the length and width of the single patch element, respectively. L_F_ and W_F_ are the length and width of the single antenna element feedline, respectively. Notably, all MPAs have a uniform featured gap of 19 mm. Figure 1b shows the bottom side of the proposed design. It is evident from the figure that all elements have truncated grounds with slots of rounded edges. Lg and Wg characterize the parameters of partial grounds with slots of dimensions S_L_ and S_W_. To provide the same common reference for the input signal with effective isolation performance between MIMO antenna elements, a whirligig structure was introduced meticulously at the bottom of the substrate. For port isolation, bandwidth enhancement, and mutual impedance matching improvement, two hook-shaped strips of 1 mm width were uniformly placed between the MIMO elements. The dimensions of the hook-shaped structure are characterized by L_U_ and W_U_. This technique extends the operating band to a lower frequency range, thus making the design compact by reducing the electrical length. All numerical simulations were carried out using CST (Computer Simulation Technology) Microwave Studio 2021. Table 1 lists all the optimized parameters of the proposed design.

### Configuration of UWB Antenna

**Figure 2:**
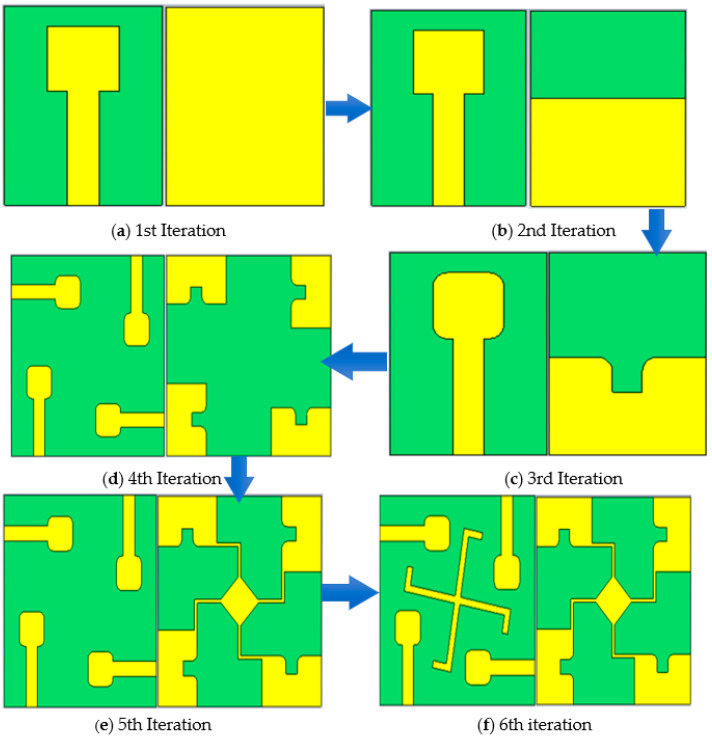
Proposed design step of single-antenna element: (**a**) full ground plane; (**b**) partial ground; (**c**) partial ground with slot; (**d**) proposed design; (**e**) connected ground; and (**f**) cross hook-shaped strips.

**Figure 3:**
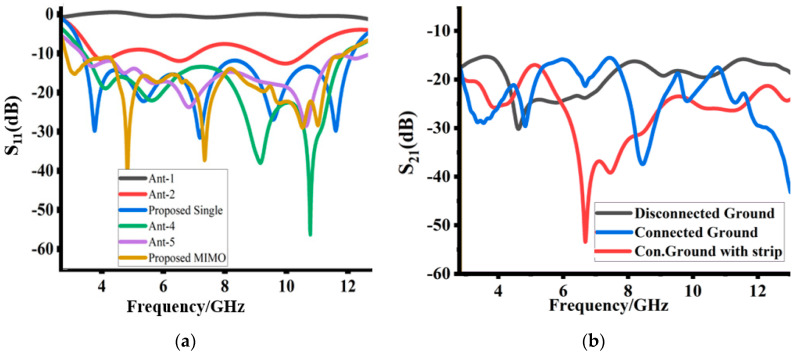
Simulation of S-parameters of different design steps: (**a**) S_11_ and (**b**) isolation co-efficient.

The detailed evaluation steps of the proposed design are shown in Figure 2a–f, and their corresponding simulated reflection co-efficients are described in Figure 3a,b. The proposed design is finalized in six steps. Each design step corresponds to certain changes in the simulated results (S-parameters). The design evaluation starts with the design of a unit element. As shown in Figure 2a, the initial design consists of a square microstrip patch antenna with a full ground plane. Figure 3a shows the corresponding reflection co-efficient of the first iteration. At this stage, the design has poor impedance matching, thus, more power is reflected and less power is transmitted. In the second iteration, the ground plane is truncated, which is shown in Figure 2b. Given in Figure 3a, at this stage the design has certain improvements in impedance matching. However, it still needs improvement. To further enhance the impedance matching, a rectangular slot is etched in the truncated ground plane. Moreover, the radiator and ground slot edges are rounded. Figure 3a shows that this iteration further enhances the impedance matching. At this stage, the single unit covers the majority of the UWB band. In the fourth iteration, the design is enhanced to include four elements of MIMO design. As shown in Figure 2d, all four symmetrical MIMO elements are positioned orthogonally. Figure 3b shows that this specific arrangement of MIMO elements gives good port isolation. Although the design has low mutual coupling between the antenna elements, the drawback is the disconnected ground. For reference, the ground plane is connected with a whirligig structure. This reduces the port’s isolation at a low frequency, which is shown in Figure 3b. To further enhance the port isolation and impedance bandwidth, cross hook-shaped strips are added symmetrically among the MIMO elements. This is shown in Figure 2f. This iteration improves the port isolation and increases the operating bandwidth to 2.8 GHz. Thus, the final step enhances the operating bandwidth as well as improves the port isolation.

## 3. Parametric Analysis of Single UWB

The proposed design starts with the design of a single-antenna element. The unit cell design is based on certain important parameters, which include the variation in the ground plane slot length and width, impact of rounding the radiator and ground slot edges, and impact of the cross hook-shaped strips. Variation in these parameters highly influences the impedance matching and operating bandwidth. A detailed parametric study is discussed below.

### 3.1. Impact of Ground Plane

**Figure 4:**
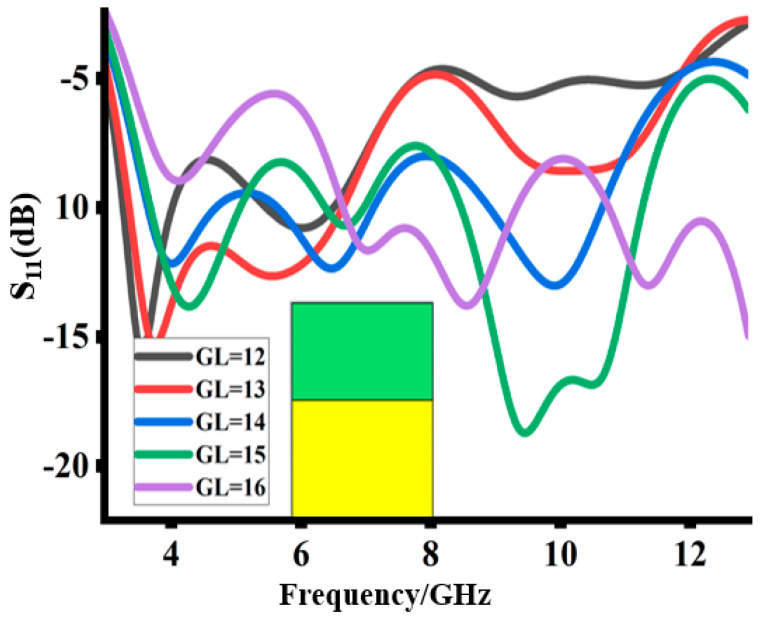
Impact of partial ground length on the reflection coefficient.

The parametric study starts by analyzing the impact of the ground plane on the reflection coefficients, as depicted in Figure 4. Gradually increasing the ground plane length “G_L_” improves the impedance matching and enhances the operating bandwidth. At the ground length of 14 mm, the maximum operating band is achieved. At this length, not only is the bandwidth at its maximum, but there is good impedance matching of the whole operating band. Further increasing the ground length deteriorates the impedance matching at higher frequencies, thus reducing the overall bandwidth.

### 3.2. Impact of Ground Slot Width and Length

**Figure 5:**
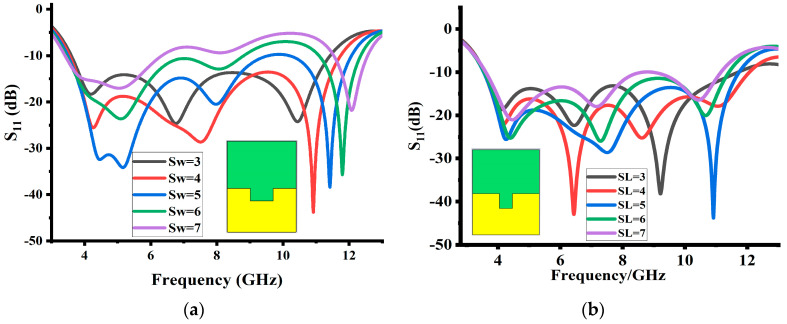
Simulated results of (**a**) impact of ground slot width and (**b**) impact of ground slot length.

The width of the ground slot is another important parameter that impacts the operating bandwidth and impedance matching. Figure 5a shows the overall impact of the ground slot width on the reflection coefficient. Gradually increasing the ground slot width “S_W_” improves the impedance matching and operating bandwidth. At the value of 4 mm, the design attains a good impedance matching and bandwidth. Further increasing the ground slot width reduces the impedance matching; thus, a slot width of 4 mm is finally selected. The impact of slot length “S_L_” on the reflection coefficients is seen in Figure 5b. The reflection coefficients graph shows that changing the length of the slot (S_L_) has an impact on the operating band and impedance matching. By increasing slot length, the impedance bandwidth improves throughout the operating band. A slot length of 5 mm is finalized. Any further increase in the slot length reduces the operating bandwidth and decreases the impedance matching.

### 3.3. Impact of Rounding Radiator and Ground Slot Edges

**Figure 6:**
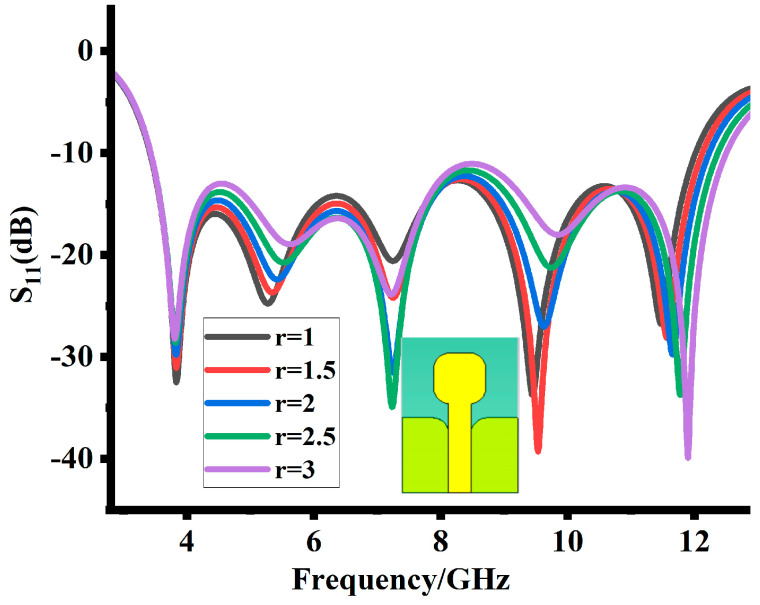
Impact of rounding edges of the radiator and ground slot.

Rounding the edges of the radiator and ground slot also impacts the impedance matching. Gradually increasing the curve increases the impedance matching. With the curve of a 2 mm radius, a good impedance matching for an overall operating band is achieved, as shown in Figure 6.

### 3.4. Impact of Cross Hook-Shaped Strip

**Figure 7:**
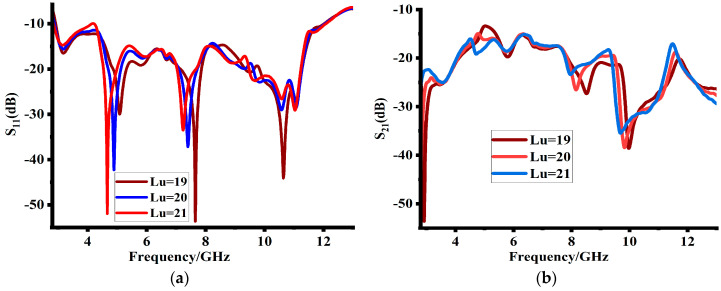
Impact of cross-shaped patch length (**a**) on reflection coefficients and (**b**) mutual impedances.

**Figure 8:**
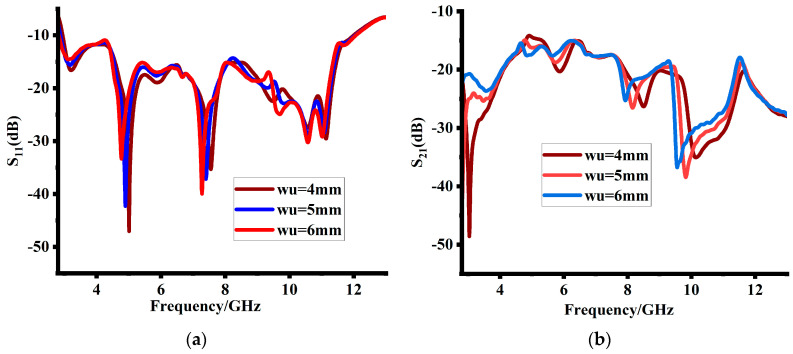
Impact of cross-shaped patch bent length (**a**) on reflection coefficients and (**b**) mutual impedances.

The cross hook-shaped strips were placed symmetrically among the MIMO antenna elements. The length and width of the cross hook-shaped patches impact both the reflection coefficients and mutual impedances. Figure 7 shows that increasing their length has minimal impact on the reflection coefficients. However, it increases the port isolation. For good performance, 40 mm is the final length value of the cross hook-shaped patch. Similarly, cross-shaped bent patch also impacts impedance matching and mutual coupling, as depicted in Figure 8. It enhances the impedance matching at lower frequencies and likewise improves the mutual coupling at the same range. A patch bent of 5 mm length was finally selected, which resulted in good impedance matching and mutual coupling, as shown in Figure 8b.

## 4. Results and Discussion

The final simulation and measurement results of the proposed design are presented in this section. These results provide details of the proposed design performance. The main performance parameters are reflection coefficients, isolation co-efficient, current distribution, far-field radiation pattern, and gain of the design.

### 4.1. Experimental Setup

With the help of the CST Microwaves Studio 2021 software, the proposed UWB-MIMO system was meticulously designed, simulated, and analyzed. To accurately describe and evaluate the antenna system’s performance, this robust electromagnetic tool was utilized. In order to verify the accuracy of the simulated results, the design was fabricated and experimental measurements were conducted. For the accurate measurement of the antenna far-field pattern, an anechoic chamber was utilized, which eliminates external reflections and interference. A Vector Network Analyzer (VNA) was used to carry out these measurements, which allows for the accurate characterization of the key parameters, including reflection coefficient and gain. During the testing process of the suggested design, Port-1 was connected with input, are other ports were terminated with 50 Ω impedance to ensure proper impedance matching, allowing for reliable and consistent measurements.

### 4.2. S-Parameters

**Figure 9:**
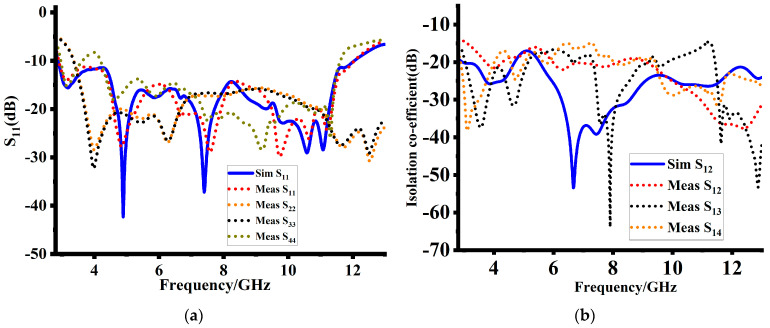
Simulated and measured results: (**a**) reflection co-efficient and (**b**) isolation co-efficient.

As indicated in Figure 9, simulations and measurements were used to evaluate the reflection coefficients of the suggested design. Overall, the simulated and measured results shows a high degree of agreement, demonstrating design validity. However, some minimal discrepancies were noticed in impedance matching at higher bands. These discrepancies can be credited to fabrication errors and deficiencies in the measuring environment. Additionally, as shown in Figure 9a, a slight difference in the measured reflection coefficient is observed, which may be affected by the different noise presences, loose connections, and inaccurate design orientation. Interestingly the measured reflection coefficients cover the same operating band as that of the simulated values. Despite these discrepancies, the overall consistency between the simulated and measured reflection coefficients validates the design’s performance. The simulated and measured mutual impedances of the suggested design are depicted in Figure 9. The measured mutual impedances are in close agreement with the simulated results, validating the design accuracy. However, at 7.8 GHz, an optimum mismatch are noticed between the simulated and measured results, indicating the imperfections in fabrication. It is important to note that the exact fabrication of antennas that have complex structures can be challenging, leading to minor deviations in performance. However, the close proximity between the simulated and measured mutual impedances speaks to the effectiveness of the proposed design. Table 2 provides a comprehensive detail of the simulated and measured reflection co-efficient.

### 4.3. Surface Current

**Figure 10:**
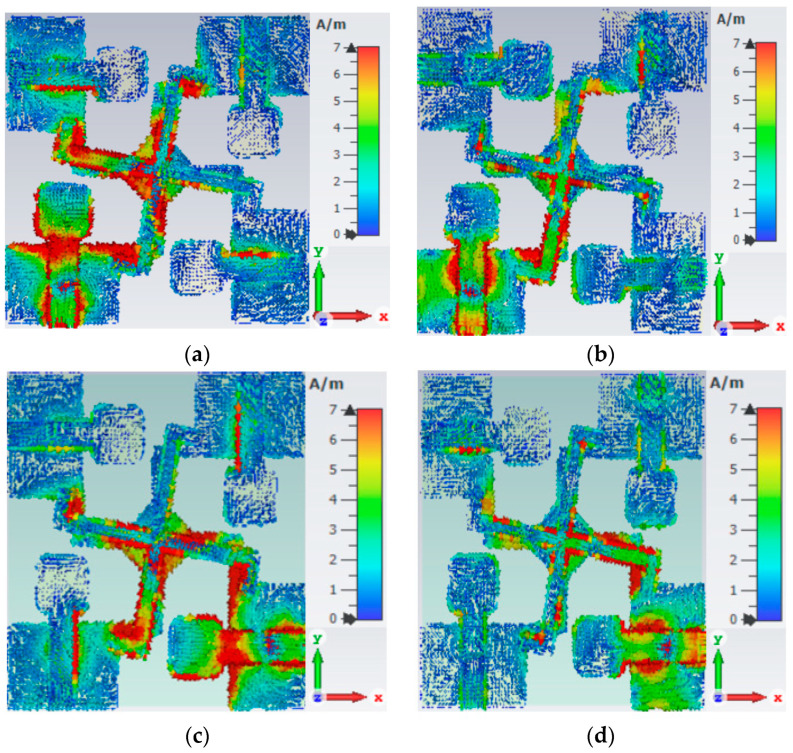
Surface current distribution of proposed 4-port UWB-MIMO antenna: (**a**) Port-1 at5 GHz; (**b**) Port-1 at 7 GHz; (**c**) Port-2 at 5 GHz; and (**d**) Port-2 at 7 GHz.

The current distribution provides insight into behavior and operating modes at the target resonance frequencies of the proposed antenna. Figure 10a shows the current distribution at 5 GHz when only Port 1 is excited while the other ports are terminated with 50 Ω. At this frequency, the current is mainly distributed along the feed line and outer part of the radiator. The current is also focused on the left side of the decoupling line, thus permitting a very low current to Port 2. This helps to ensure maximum port isolation while keeping the mutual impedance minimum. Figure 10b shows the current distribution for the same port at 7 GHz. At this frequency, the current exhibits distribution along the feed line when the lower part of the radiator is connected with it. Figure 10c,d shows the distribution of the current when Port 2 is active and all the other ports are terminated with 50 Ω. For Port 2, the current distribution at both resonance frequencies repeats the same pattern, ensuring good impedance matching and port isolation.

### 4.4. Radiation Characteristics

**Figure 11:**
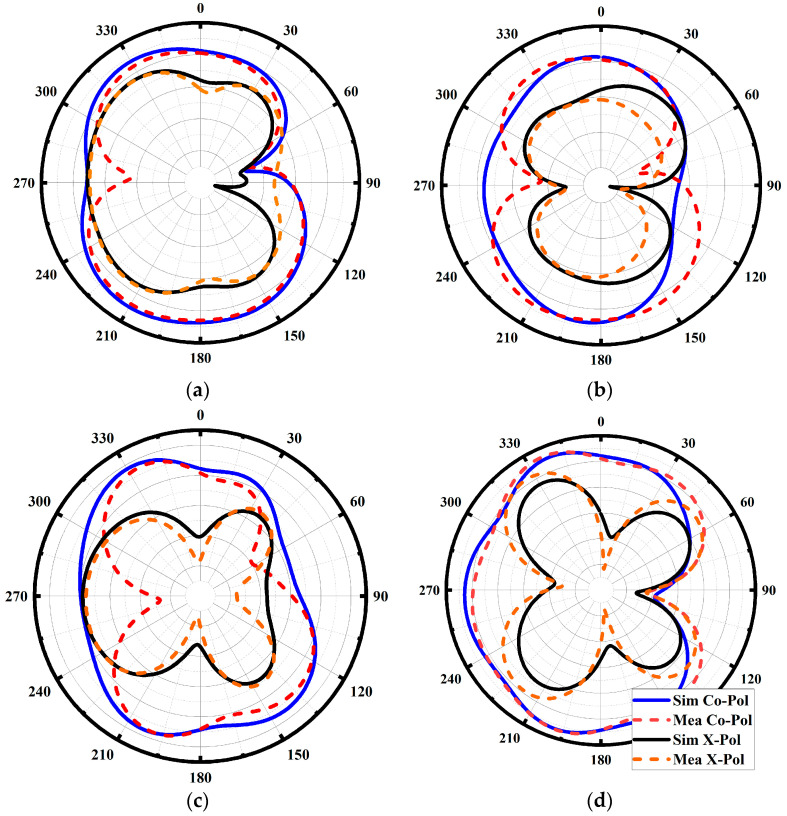
Simulated and measured 2D radiation pattern using Por-1 (**a**) E-Field at 5 GHz; (**b**) H-field at 5 GHz; (**c**) E-Field at 7 GHz; and (**d**) H-field at 7 GHz.

The proposed design’s electrical (E) (*XY*-plane) and magnetic (H) (*XZ*-plane) far-field radiation patterns at the achieved resonance frequencies are shown in Figure 11a–d. By activating Port 1 and using a 50-ohm load to terminate the remaining ports, the radiation patterns were obtained. The radiation patterns for both ports show good agreement between simulation and measurement, with an omnidirectional pattern and focused toward 330°. Every port displays identical radiation patterns, indicating a uniform performance through the entire band. Peak gain for both ports ranged from 5 to 6.8 dBi for co-polarization and from −9 to −10 dBi for cross-polarization, indicating that the antenna’s radiated power is focused in the desired direction, although some power is also radiated in other directions. In a multi-path scenario, this value of cross-polarization is not the real matter of concern. It should be emphasized that small discrepancies between the simulated and measured far-field radiation patterns at 5 GHz and 7 GHz can be attributable to human and experimental error. As a result of these considerations, some minor discrepancies at particular frequencies are introduced.

### 4.5. Simulated and Measured Gain

**Figure 12:**
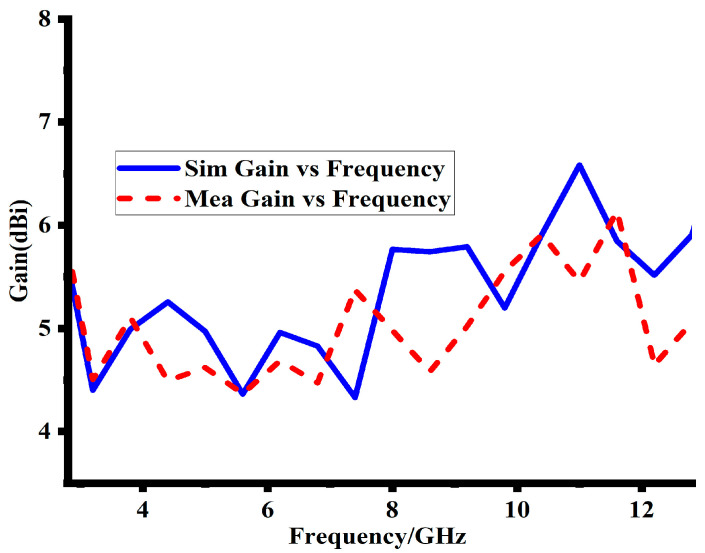
Simulated and measured gain of the proposed MIMO design at Port-1.

**Figure 13:**
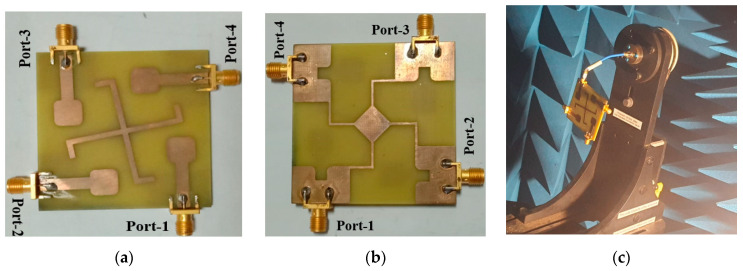
Proposed design setup: (**a**) front view; (**b**) bottom view; and (**c**) measurement setup.

Antenna performance is determined by its gain, which also helps to determine its ability to efficiently radiate power over the entire operating band. The gain of the suggested design through the entire band is calculated through simulation and measurement, as given in Figure 12. The designs measured gain ranges from 5 to 6.8 dBi. At both the lower and higher operating bands, the measured gain values nearly matches the simulated results. However, a slight variation is seen at the mid-range frequencies. This deviation may be due to numerous factors, including counting fabrication errors and environmental noise. Despite this minor discrepancy, the simulated and measured gains match, showing how well the proposed design leads to the desired radiation features. The details of the simulated and measured achieved gain are depicted in Table 3. Figure 13a,b shows the top and bottom views of the fabricated prototype, respectively. Figure 13c depicts the fabricated prototype placed within the measuring facility for far-field radiation pattern measurements, ensuring the accurate evaluation of the antenna’s radiation characteristics.

## 5. MIMO Parameters

Important factors like the envelope correlation coefficient (ECC), diversity gain (DG), channel capacity loss (CCL), mean effective gain (MEG), and total active reflection coefficient (TARC) were analyzed to evaluate the MIMO and diversity performance of the proposed antenna.

### 5.1. Envelop Correlation Coefficient

**Figure 14:**
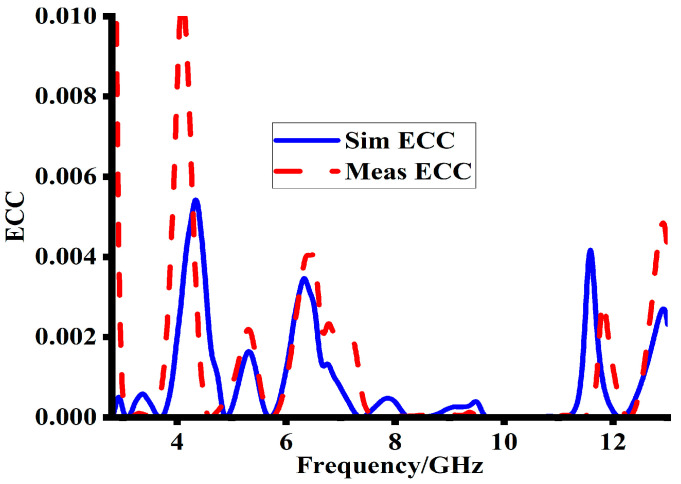
Simulated and measured ECC results of the proposed MIMO antenna.

ECC measures the correlation or isolation between the various branches of communication; the ECC is a key parameter in MIMO antenna systems. In this study, the suggested MIMO antenna’s far-field patterns were used to compute the ECC, which is represented using Equation (1) [17]. In Equation (1), $\rho_{e}$ is the ECC, XPR is the power discrimination ratio of the vertical and horizontal components of the radiation pattern, $E_{\theta 1}$, $E_{\theta 2}^{*}$ and $E_{\varphi 1},{\;E}_{\varphi 2}^{*}$ are the electric field components in elevation and azimuth directions, respectively, and *P_θ_* and *P_φ_* are the angular power in elevation and azimuth directions, respectively [18]. A suitable ECC value for adequate MIMO performance is often less than 0.5 [19]. The simulated and measured ECC of the proposed MIMO antenna is Ihown in Figure 14. The results show that the ECC is substantially below 0.01 in the targeted frequency bands. In order to achieve high-quality MIMO communication, the antenna branches whose alternative representation is low ECC must be effectively isolated.

(1) $$\rho_{e}=\frac{|\int_{0}^{2\pi }\int_{0}^{\pi }(XPR.\;E_{\theta 1}.E_{\theta 2}^{*}.P_{\theta }+E_{\varphi 1}.\;E_{\varphi 2}^{*}.P_{\varphi })d\Omega {|}^{2}}{\int_{0}^{2\pi }\int_{0}^{\pi }(XPR.\;E_{\theta 1}.E_{\theta 1}^{*}.P_{\theta }+E_{\varphi 1}.\;E_{\varphi 2}^{*}.P_{\varphi })d\Omega \times \int_{0}^{2\pi }\int_{0}^{\pi }(XPR.\;E_{\theta 2}.E_{\theta 2}^{*}.P_{\theta }+E_{\varphi 2}.\;E_{\varphi 2}^{*}.P_{\varphi })d\Omega }$$

### 5.2. Diversity Gain (DG)

**Figure 15:**
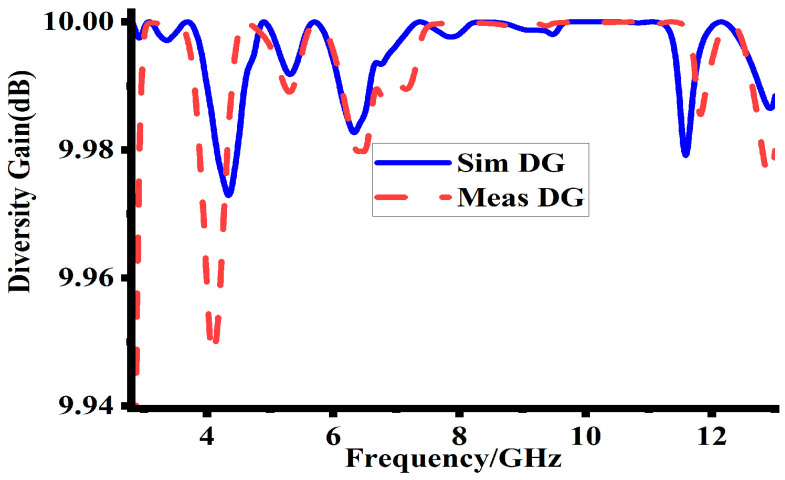
The simulated and measured DG of the proposed design.

Another key variable used to compare the usefulness of MIMO antenna systems to single-antenna systems is diversity gain (DG). Equation (2) is used to calculate DG and measures the strategies to increase the signal quality [21]. In Equation (2), DG is the diversity gain of the design, and ECC is the envelop correlation coefficient of the design. Thus, DG is highly ECC dependent. The simulated and measured DG of the suggested MIMO design are depicted in Figure 15. The results indicate that DG is approximately 10 dB throughout the band. This high value of DG indicates the tremendous performance of the proposed MIMO antenna system. The attained DG demonstrates how well the suggested antenna design works to increase signal reliability in MIMO communication environments. Table 4 gives detailed information on the suggested antenna’s MIMO performance (ECC, DG).

(2) $$DG=10\sqrt{\left(1-{ECC}^{2}\right)}$$

### 5.3. Channel Capacity Loss (CCL)

Chanel Capacity Loss (CCL) is another vital MIMO antenna parameter. The effectiveness of the suggested design throughput can be demonstrated with the help of CCL. The better the data transfer, the lower the CCL value should be. A desirable CCL value for data transmission is 0.4 bits/s/Hz [22]. Equations (3)–(5) illustrate the fundamental variables that influence how the suggested design will ultimately respond to the CCL [23].

(3) $$C\left(Loss\right)=-\log_{2}det(\psi^{R})$$

where $\psi^{R}$ is the correlation matrix of the receiving antenna.

(4) $$\psi^{R}=\left[\begin{array}{cc} \rho 11 & \rho 12\\ \rho 21 & \rho 22 \end{array}\right]$$

(5) $$\rho \mathrm{ii}=-\left(Sii*Sij+Sjj*SijI\right)$$

Whereas    ρii=1−I, for i, j=1 or 2

**Figure 16:**
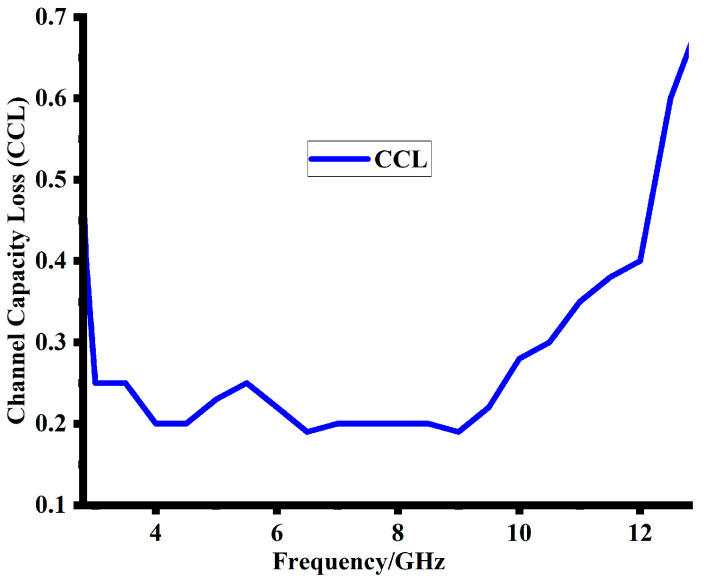
Simulated Channel Capacity Loss (CCL) of the proposed design.

Sii and Sij are the scattering parameters matrix. Figure 16 demonstrates that this design maintained a CCL value of less than 0.3 bits/s/Hz at the lower frequency band and 0.35 bits/s/Hz at the higher frequency band. This CCL value demonstrates an efficient transmission of data and throughput.

### 5.4. Mean Effecive Gain (MEG)

**Figure 17:**
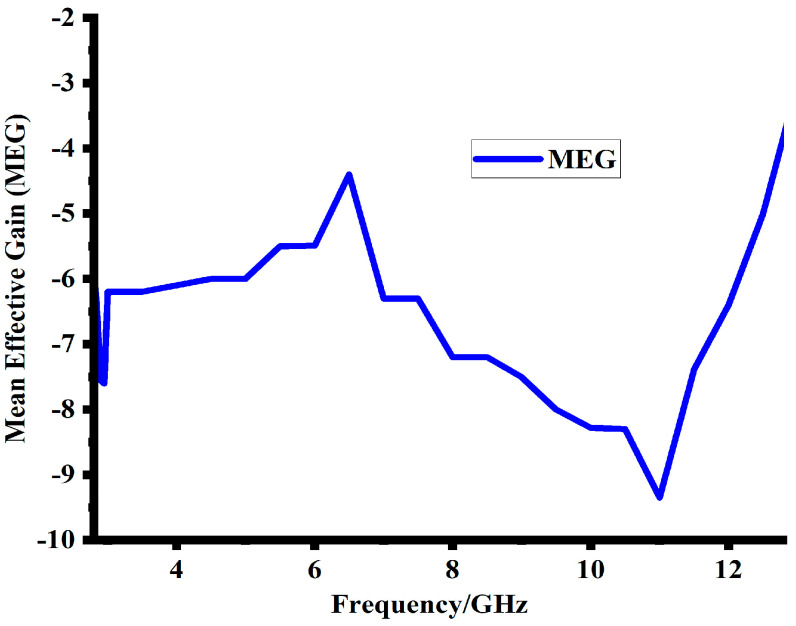
Simulated mean effective gain (MEG) of the proposed design.

Another key component in the design of MIMO antennas is mean effective gain (MEG). It is the amount of power received when comparing diversity antennas to isotropic antennas. It demonstrates how the antenna can absorb electromagnetic radiation in a multi-path environment. Using Equations (6) and (7), MEG is calculated [23]. In Equations (6) and (7), Sii, Sij, and Sjj represent the scattering parameters matrix [24]. The MEG among the antenna ports must be less than 3 dB for optimal performance. As shown in Figure 17, the value of MEG is less than 4.5 dB throughout the operating band of the suggested design.

MEGi = 0.5[1 − |Sii|^2^ − |Sii|^2^] (6)

MEGi = 0.5[1 − |Sij|^2^ − |Sjj|^2^] (7)

### 5.5. Tottal Active Reflaction Co-Efficient

Another key factor of the MIMO antenna is the Total Active Reflection Coefficient (TARC). It is the proportion between the square root of the total power incident and reflected. It is useful in finding out the operational bandwidth of MIMO antenna systems. For a MIMO antenna system, the TARC is determined using Equation (8). In Equation (8), the TARC is determined using the S-parameters (*S_ik_*) and phase angle (ɵ).

(8) $$\mathrm{TARC}=N^{-0.5}*\sqrt{\sum_{i=1}^{N}{\left|\sum_{k=i}^{N}s_{{ik}^{e^{j\theta }k-1}}\right|}^{2}}$$

**Figure 18:**
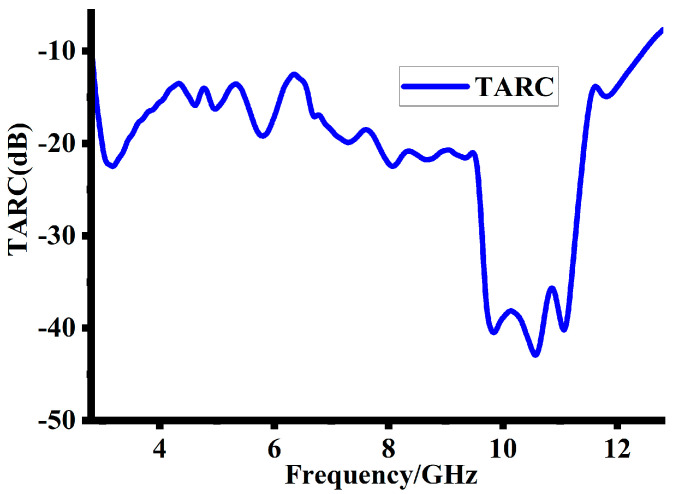
Simulated TARC of the proposed design.

A MIMO antenna system’s TARC should be below −10 dB. Therefore, any TARC value below −10 dB is seen to be optimal for improved communication. Figure 18 demonstrates that the suggested design has a TARC less than −10 dB through the entire bandwidth. For the suggested design, this value of TARC is acceptable for the transmitting and receiving ends.

### 5.6. Simulated Time Domain Analysis

**Figure 19:**
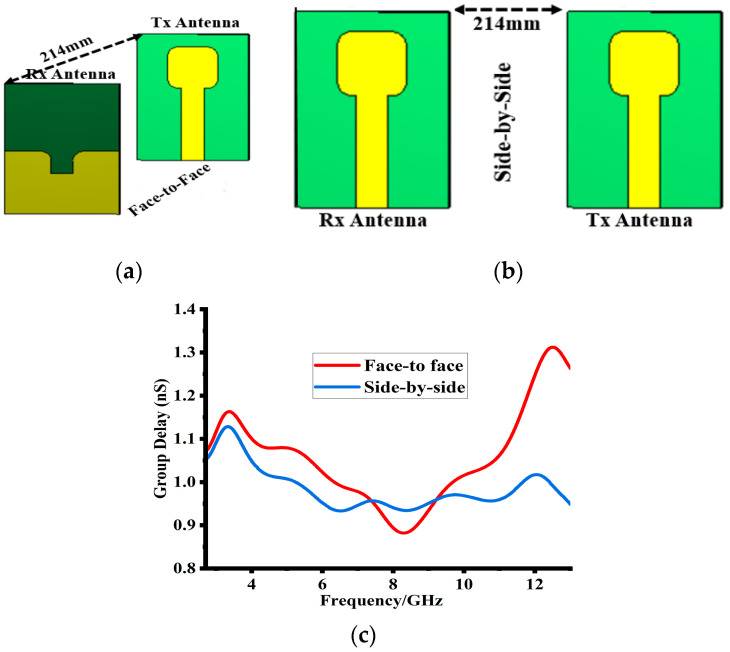
(**a**,**b**) shows the design orientations for group delay determination. (**c**) Shows the simulated results of the calculated group delay for both orientations.

Group delay is another important parameter of UWB antenna design performance. It describes the amount of delay introduced in frequencies while passing through different components of the devices. A large group delay shows the distortion in the transmitted signal. The group delay of the proposed design was determined in two orientations. Figure 19a,b shows two orientations: face-to-face and side-by-side. For both orientations, the designs were isolated at a distance of 214 mm (2 times λ0 at 2.8 GHz) to create a far-field atmosphere. As shown in Figure 19a,b, ant-1 acted as Tx (transmitter) and ant-2 acted as Rx (Receiver). Figure 19a shows that face-to-face orientation has a maximum group delay of 1.3 ns, whereas Figure 19b shows a side-by-side group delay of 1.1 ns. Based on the achieved results, it is concluded that the time-domain behavior of the proposed design has linear transmission properties with minimal group delay throughout the operating band.

Table 5 demonstrates a detailed comparison of the suggested design with some recently published works. The comparison emphasizes on numerous essential parameters, including electrical and physical size, operating bands, peak gain, envelope correlation coefficient (ECC), diversity gain (DG), and isolation among ports. Based on the comparison, it is clear that this work is compact (miniaturized) in size while giving excellent performance. The antenna design attains compact electrical and physical dimensions in comparison to the published work, showing its potential to easily house in the devices designed for UWB wireless applications. In addition to this, the suggested design exhibits a wider operating band with better impedance matching, maximum gain, ECC, and DG and has maintained high port isolation with simple decoupling techniques. This demonstrates the effectiveness and competitiveness of the suggested antenna design in terms of size, performance, and MIMO capabilities (envelop correlation coefficient of 0.003, DG of 10 dB, and over 20 dB isolation). Moreover, the inclusion of a crossed-shaped structure on the substrate greatly improved mutual impedance, resulting in better isolation between the MIMO elements. The measured results closely align with the simulated results, confirming the validity of the prototype for the target wireless application.

## 6. Conclusions

A novel 4-port UWB-MIMO antenna was successfully designed, simulated, and fabricated. The suggested UWB-MIMO antenna system demonstrates outstanding performance for wireless applications. The antenna elements were arranged symmetrically in an orthogonal manner for good diversity polarization. Two different techniques were introduced to enhance the performance. For the provision of the same reference signal, a whirligig structure was introduced in the ground plane. The impedance matching at lower frequencies and port isolation were effectively enhanced by incorporating an inverted cross hook-shaped strip on the top of a substrate. The UWB-MIMO design demonstrates excellent diversity parameters, including a maximum DG of 10 dB, an ECC of 0.003, TARC less than −10 db, MEG better than −3 Db, CCL less than 0.4 bits/s/Hz, and over 20 dB port isolation. The fabricated prototype measurements are in close agreement with the simulated results, verifying the robustness and viability of the UWB-MIMO antenna design.

## Figures and Tables

**Table 1:** Different parameters of proposed single-antenna element.

Parameters	Dimensions (mm)	Parameters	Dimensions (mm)	Parameters	Dimensions (mm)
m_t_	0.035	W_P_	9.5	S_L_	29
H_S_	1.6	L_P_	9.5	L_U_	40
r	2	G_L_	14	W_F_	4.23
S_W_	4	L_F_	16.6	L_S_	58
S_L_	5	S_W_	21	W_S_	58
W_U_	5	X	19	a	10
b	9	c	8.5	d	1

**Table 2:** Details of simulated and measured S-parameters.

Sr. No	Resonance Freq (fr) (GHz)	Sim. S_11_	Meas. S_11_	Simulated S_12_	Meas. S_12_
1	5	−42	−27	−18	−19
2	7	−38	−29	−19	−21

**Table 3:** Details of simulated measured gain.

Sr. No	Resonance Freq. (GHz)	Sim. Gain (dB)	Meas. Gain (dB)
1	5	5	4.75
2	7	4.66	4.56

**Table 4:** Details of simulated and measured ECC and DG.

Sr.No	Resonance Freq. (GHz)	Sim. ECC	Mea. ECC	Sim. DG	Mea. DG
1	5	0.0002	<0.002	9.998	9.9
2	7	0.0007	0.002	9.998	9.98

**Table 5:** Comparison of the proposed design with recently published works.

Ref.	Elements	Common Ground	Freq (GHz)	Size(mm^2^)/λ^2^*_g_*	Gain (dB)	ECC/DG	Isolation (dB)
[24]	4	No	3.5–11	54 × 54/ (0.63 × 0.63)	--	>0.03/--	>17
[25]	4	No	3–10.86	65 × 65/ (0.65 × 0.65)	--	0.01/9	>22
[26]	4	No	4.89–5.61	70 × 70/ (1.14 × 1.14)	4.0	0.3/9.9	22
[27]	4	No	4.71–6.62	45 × 45/ (0.7 × 0.7)	4.95	0.2/--	20
[28]	2	Yes	4.18–6.58	80 × 50/ (1.11 × 0.69)	4	0.056/9.8	17
[29]	4	Yes	2.2–6.28	45 × 45/ (0.3 × 0.3)	3	0.25/--	<14
[30]	4	Yes	2–6	110 × 114/ (0.73 × 0.76)	4.8–37.5	------	<15
[31]	4	No	3.5–11	62.5 × 60.5/ (0.72λ × 0.70λ)	4	0.01/9.95	>20
[32]	4	Yes	8.5–16	78 × 78/ (2.2λ × 2.2λ)	5.1	0.005/-	>20
**Proposed**	4	Yes	2.8–12.1	58 × 58/ (0.5λ × 0.5λ)	>5.96	>0.003/9.99	>20

## Data Availability

The data will be available on request.

## References

[1] Rappaport T.S., Sun S., Mayzus R., Zhao H., Azar Y., Wang K., Wong G.N., Schulz J.K., Samimi M., Gutierrez F. (2013). Millimeter wave mobile communications for 5G cellular: It will work!. IEEE Access.

[2] Hasan M.M., Faruque M.R.I., Islam M.T. (2018). Dual band metamaterial antenna for LTE/bluetooth/WiMAX system. Sci. Rep..

[3] Abbas M.A., Allam A., Gaafar A., Elhennawy H.M., Sree M.F.A. (2023). Compact UWB MIMO antenna for 5G millimeter-wave applications. Sensors.

[4] Abbas A., Hussain N., Jeong M.-J., Park J., Shin K.S., Kim T., Kim N. (2020). A rectangular notch-band UWB antenna with controllable notched bandwidth and centre frequency. Sensors.

[5] Amin F., Saleem R., Shabbir T., Rehman S.U., Bilal M., Shafique M.F. (2019). A compact quad-element UWB-MIMO antenna system with parasitic decoupling mechanism. Appl. Sci..

[6] Nadeem I., Choi D.-Y. (2018). Study on mutual coupling reduction technique for MIMO antennas. IEEE Access.

[7] Haq M.A.U., Koziel S.J. (2018). Ground plane alterations for design of high-isolation compact wideband MIMO antenna. IEEE Access.

[8] Iqbal A., Saraereh O.A., Ahmad A.W., Bashir S. (2017). Mutual coupling reduction using F-shaped stubs in UWB-MIMO antenna. IEEE Access.

[9] Yin Y., Hong J., Luo C., Amin M. (2017). A compact planar UWB MIMO antenna using modified ground stub structure. IEICE Electron. Express.

[10] Qamar Z., Riaz L., Chongcheawchamnan M., Khan S.A., Shafique M.F. (2014). Antennas; Propagation. Slot combined complementary split ring resonators for mutual coupling suppression in microstrip phased arrays. IET Microw. Antennas Propag..

[11] Wu D., Cheung S.W., Li Q.L., Yuk T.I. (2017). Antennas; Propagation. Decoupling using diamond-shaped patterned ground resonator for small MIMO antennas. IET Microw. Antennas Propag..

[12] Gogosh N., Shafique M.F., Saleem R., Usman I., Faiz A. (2013). An UWB diversity antenna array with a novel H-type decoupling structure. Microw. Opt. Technol. Lett..

[13] Srivastava G., Mohan A. (2015). Compact MIMO slot antenna for UWB applications. IEEE Antennas Wirel. Propag. Lett..

[14] Tang Z., Wu X., Zhan J., Hu S., Xi Z., Liu Y. (2019). Compact UWB-MIMO antenna with high isolation and triple band-notched characteristics. IEEE Access.

[15] Tao J., Feng Q. (2016). Compact ultrawideband MIMO antenna with half-slot structure. IEEE Antennas Wirel. Propag. Lett..

[16] Yadav D., Abegaonkar M.P., Koul S.K., Tiwari V.N., Bhatnagar D. (2018). Two element band-notched UWB MIMO antenna with high and uniform isolation. Prog. Electromagn. Res. M.

[17] Mathur R., Dwari S.J.F. (2018). Compact 4-Port MIMO/diversity antenna with low correlation for UWB application. Frequenz.

[18] Wu W., Yuan B., Wu A. (2018). Propagation. A quad-element UWB-MIMO antenna with band-notch and reduced mutual coupling based on EBG structures. Int. J. Antennas Propag..

[19] Anitha R., Vinesh P., Prakash K., Mohanan P., Vasudevan K. (2016). Propagation. A compact quad element slotted ground wideband antenna for MIMO applications. IEEE Trans. Antennas Propag..

[20] Ibrahim A.A., Abdalla M.A., Hu Z. (2017). Design of a compact mimo antenna with asymmetric coplanar strip-fed for UWB applications. Microw. Opt. Technol. Lett..

[21] Wu A., Zhao M., Zhang P., Zhang Z. (2022). A compact four-port MIMO antenna for UWB applications. Sensors.

[22] Babu K.V., Anuradha B. (2019). Design of MIMO antenna to interference inherent for ultra wide band systems using defected ground structure. Microw. Opt. Technol. Lett..

[23] Sree G.N.J., Nelaturi S. (2021). Opportunistic control of fractal-based MIMO antenna for sub-6-GHz 5G applications. Int. J. Commun. Syst..

[24] Sharawi M.S. (2017). Current misuses and future prospects for printed multiple-input, multiple-output antenna systems [wireless corner]. IEEE Antennas Propag. Mag..

[25] Ibrahim A.A., Ahmed M.I., Ahmed M.F. (2022). A systematic investigation of four ports MIMO antenna depending on flexible material for UWB networks. Sci. Rep..

[26] Zhang J., Du C., Wang R. (2022). Design of a four-port flexible UWB-MIMO antenna with high isolation for wearable and IoT applications. Micromachines.

[27] Malviya L., Panigrahi R.K., Kartikeyan M. (2016). A 2 × 2 dual-band MIMO antenna with polarization diversity for wireless applications. Prog. Electromagn. Res. C.

[28] Saadh A.M., Ramaswamy P., Ali T. (2021). A CPW fed two and four element antenna with reduced mutual coupling between the antenna elements for wireless applications. Appl. Phys. A.

[29] Jehangir S.S., Sharawi M.S. (2017). A miniaturized UWB biplanar Yagi-like MIMO antenna system. IEEE Antennas Wirel. Propag. Lett..

[30] Yu K., Li Y., Liu X. (2018). Mutual coupling reduction of a MIMO antenna array using 3-D novel meta-material structures. Appl. Comput. Electromagn. Soc. J. (ACES).

[31] Abdelghany M.A., Sree M.F.A., Desai A., Ibrahim A.A. (2023). 4-Port Octagonal Shaped MIMO Antenna with Low Mutual Coupling for UWB Applications. CMES-Comput. Model. Eng. Sci..

[32] El-Gendy M.S., Ali M.M.M., Thompson E.B., Ashraf I. (2023). Triple-Band Notched Ultra-Wideband Microstrip MIMO Antenna with Bluetooth Band. Sensors.

